# Estimating the number of contributors to two-, three-, and four-person mixtures containing DNA in high template and low template amounts

**DOI:** 10.3325/cmj.2011.52.314

**Published:** 2011-06

**Authors:** Jaheida Perez, Adele A. Mitchell, Nubia Ducasse, Jeannie Tamariz, Theresa Caragine

**Affiliations:** Office of Chief Medical Examiner of the City of New York, The Department of Forensic Biology, New York, NY, USA

## Abstract

**Aim:**

To develop guidelines to estimate the number of contributors to two-, three-, and four-person mixtures containing either high template DNA (HT-DNA) or low template DNA (LT-DNA) amounts.

**Methods:**

Seven hundred and twenty-eight purposeful two-, three-, and four-person mixtures composed of 85 individuals of various ethnicities with template amounts ranging from 10 to 500 pg were examined. The number of alleles labeled at each locus and the number of labeled different and repeating alleles at each locus as well over all loci for 2 HT-DNA or 3 LT-DNA replicates were determined. Guidelines based on these data were then evaluated with 117 mixtures generated from items handled by known individuals.

**Results:**

The number of different alleles over all loci and replicates was used to initially categorize mixtures. Ranges were established based on the averages plus and minus 2 standard deviations, and to encompass all observations, the maximum and the minimum values. To differentiate samples that could be classified in more than one grouping, the number of loci with 4 or more repeating or different alleles, which were specific to three- and four-person mixtures, were verified. Misclassified samples showed an extraordinary amount of allele sharing or stutter.

**Conclusions:**

These guidelines proved to be useful tools to distinguish low template and high template two-, three-, and four-person mixtures. Due to the inherent higher probability of allele sharing, four-person mixtures were more challenging. Because of allelic drop-out, this was also the case for samples with very low amounts of template DNA or extreme mixture ratios.

Interpretation of DNA mixtures derived from crime scene evidence samples is a major challenge in forensic DNA analysis. Evidence samples are typically deemed mixtures if they demonstrate more than 2 alleles at one or more loci, although allowances may be made for stutter and other artifacts of the short tandem repeat (STR) profiling process. Peak height imbalance at heterozygous loci may also indicate a mixture. Mixtures arise when 2 or more individuals contribute to an evidence sample. Contributors to an evidence sample may include perpetrator(s), victim(s), and/or other individuals who have come into contact with the crime scene, whether connected to the crime or not.

Several different approaches can be taken to interpret DNA mixtures and to evaluate the strength of a comparison between an evidence sample and potential contributors. For some samples, the individual contributors to a mixture can be separated, or deduced (1-5) and, once separated, random match probability can be applied to the profiles of the individual contributors. For samples that cannot be deduced, one may compute a likelihood ratio or probability of exclusion in order to evaluate the strength of a comparison between a putative contributor and the mixture. Both methods are discussed in Buckleton et al (6) and Balding (7).

Likelihood ratio (LR) based methods implicitly assume that the number of contributors to a mixture is known. Before computing the LR, one must specify prosecution and defense hypotheses on which to condition in the numerator and the denominator, respectively, of the ratio. Each hypothesis contains a specified number of individuals, for example, the prosecution hypothesis may be that the sample is a mixture of DNA from a suspect and an unknown, unrelated person and the defense hypothesis may be that the sample is a mixture of DNA from 2 unknown, unrelated persons. Therefore, the first steps in the calculation of the LR are determination of the number of contributors to the mixture and specification of the mixture components under each of the competing hypotheses.

The most general formulation of the Probability of Exclusion does not require explicit specification of the number of contributors to a mixture, as no attempt is made to explain all of the alleles that are observed. Budowle et al (8) also describe a restricted Random Man Not Excluded (RMNE) approach, in which possible contributors’ genotypes are restricted by peak heights, alleles that have already been attributed to another component of the mixture, and the assumed number of contributors. Thus, to apply the restricted RMNE, one would need to determine the number of contributors to a mixture. Even when the analytical methods employed do not require specification of the number of contributors to a forensic mixture, having such an estimate may be helpful, depending on the circumstances of the case.

Interpretation guidelines from the Scientific Working Group on DNA Analysis Methods specify that the minimum number of contributors to a mixture can be determined based on the locus that exhibits the greatest number of peaks, with an allowance for tri-allelic loci and/or stutter (9). Following these allele-counting guidelines, a sample with 3or more labeled alleles at 1 or more loci can be considered to contain a minimum of 2 contributors, a sample with 5 or more labeled alleles at 1 or more loci can be considered to contain a minimum of 3 contributors, and so on. A sample may also be deemed a two-person mixture even if no loci exhibit 3 or more peaks if heterozygous loci are more imbalanced than a laboratory’s empirically determined limits.

While locus-by-locus allele counting can provide an estimate of the minimum number of contributors to a mixture, it may not indicate the actual number of contributors to mixtures, particularly those with 3 or more contributors (10,11). That is, using this method, a three-person mixture could be classified as a mixture of at least 2 people and a four-person mixture could be designated a mixture of at least 3 people and sometimes as a mixture of at least 2 people. Empirical analysis of conceptual mixtures of individuals typed at 13 loci estimated that using the maximum number of alleles observed at any locus, 3.2%-3.4% of three-person mixtures would be categorized as mixtures of at least 2 people and approximately 76% of four-person mixtures would be classified as mixtures of at least 2 or at least 3 people (10). Analysis of conceptual mixtures of simulated individuals typed at SGM+ and Profiler Plus loci gave similar results (11).

Uncertainty in the number of contributors to a mixture has unknown effects on the restricted RMNE. However, the effect on the LR of uncertainty in number of contributors has been explored. Bounding the LR has been suggested as a means to avoid anti-conservative bias when the number of contributors to a mixture is in dispute (12-15). The denominator of the LR is usually maximized by selecting the defense hypothesis with the minimum number of contributors required to explain the evidence (13). In other words, for a given prosecution hypothesis, using the defense hypothesis with the minimum possible number of contributors will usually result in the lowest possible LR, ie, the LR that most favors the defendant. Based on results of the simulations performed by Buckleton et al (11), there is “moderate risk” of a non-minimal LR when the defense hypothesis with the minimum number of contributors is used.

One could argue that a better approach than opting for the minimum number of contributors to a mixture might be to determine the number of contributors best supported by the data. Several recent publications have explored this idea using maximum likelihood to estimate the most likely number of contributors to a mixture. Egeland et al (16) proposed an estimator using diallelic markers, assuming Hardy-Weinberg equilibrium in the population of origin. Haned et al (17) extended the method to accommodate multi-allelic markers and to allow for population substructure. The predictive value of this model was assessed in Haned et al (18). The maximum likelihood method correctly estimated the number of contributors to two- and three-person mixtures more than 90% of the time, outperforming the maximum allele count method. For four- and five-person mixtures, the maximum likelihood method gave correct classifications of mixtures 64%-79% of the time, a dramatic increase in efficiency over the maximum allele count method. Success rates remained high in the presence of population substructure and when degradation of evidence samples was simulated. Although locus drop-out was considered in the degradation model, the commonly observed phenomena of random allelic drop-out and drop-in were not considered with any of the maximum likelihood or maximum allele count methods.

To complement the maximum likelihood and maximum allele count approaches, we examined 728 two-, three-, and four-person purposeful mixtures and identified characteristics that can assist in estimating the number of contributors to the mixtures. To date, only conceptual mixtures have been examined and this is the first empirically based study of this nature. To account for allelic drop-out and drop-in, template amounts ranged from 10 pg to 500 pg. Considering empirical observations unique to mixtures in different quantitative ranges and the total number of different alleles seen across all loci, a set of guidelines was developed. The guidelines were then applied to a set of 117 items handled by 2, 3, and 4 individuals.

## Materials and methods

Protocols for preparation and quality control of personnel, workspace, equipment, and consumable preparation were performed as previously described in Caragine et al (19).

### Samples

Buccal swabs and blood samples from known donors, and items handled by individuals whose DNA profiles were known, were used. Eighty-five different donors were sampled representing the diverse population of the City of New York. The race was known for 61 (72%) of the donors as they were laboratory employees. They could be categorized generally as follows: 20% Asian, 16% Black, 54% Caucasian, and 10% Hispanic. The remaining 24 or 28% of the donors represented an anonymous sampling of the City of New York, which is composed of 9.8% Asians, 26.6% Blacks, 44.7% Caucasians, and 27% Hispanics (20). Items were either cleaned or were left un-cleaned prior to handling. In total, 728 purposeful mixtures and 117 samples from items handled by 2 (n = 36), 3 (n = 45), and 4 (n = 35) persons were tested.

### Sampling and extraction

Buccal and blood specimens were extracted with the Qiagen M48 BioRobot (Qiagen, Valencia, CA, USA). Touched items were sampled with a patent pending swab from the Office of the Chief Medical Examiner of the City of New York pre-moistened with 0.01% sodium dodecyl sulfate (21). The swabs in their entirety were incubated in 0.05% sodium dodecyl sulfate and 0.72 mg/mL Proteinase K at 56°C for 30 minutes with shaking at 1400 rpm, at 99°C for 10 minutes, and at 4°C for 5 minutes without shaking. The digest was purified twice and concentrated with a Microcon^®^ 100 (Millipore, Billerica, MA, USA) (22) pretreated with 1 µg of fish sperm DNA and eluted with 20 µL of irradiated water.

### Quantitation

Two microliters of sample was measured on the Rotor-Gene Q 3000^®^ (Qiagen) using an Alu-based real time polymerase chain reaction (PCR) assay based on the method described by Nicklas and Buel (23), with the exception of the addition of 0.3 µL of 100 × SYBR green I (Molecular Probes) and 0.525 mg/mL bovine serum albumin in a 25 µL reaction volume. Prior to making purposeful mixtures, each contributor’s DNA extract was measured in triplicate, 3 times for 9 measurements and the average of these 9 measurements was used as the concentration of the sample for the purpose of setting up dilutions.

### Amplification

Samples were amplified using the AmpFlSTR^®^ Identifiler^®^ PCR Amplification Kit (Applied Biosystems, Foster City, CA, USA) following the manufacturer’s recommendations with the exception of a two-minute annealing time, a half-reaction volume, and 2.5 U of AmpliTaq Gold^®^ for either 28 (High Template DNA [HT-DNA]) or 31 (Low Template DNA [LT-DNA]) cycles. HT-DNA samples were amplified in duplicate with at least 100 pg of DNA in each replicate. LT-DNA samples were amplified in triplicate with 100 pg or less of DNA in each replicate (19).

Seven hundred and twenty-eight purposeful mixed samples were amplified. These included 355 mixtures with HT-DNA amounts, specifically 184 from 2 persons, 121 from 3 persons, and 50 from 4 persons; and 373 mixtures with LT-DNA amounts, 199 from 2 persons, 124 from 3 persons, and 50 from 4 persons. The mixture ratios varied as follows for 28 cycle samples: 1:1 (n = 50), 2:1 (n = 34), 4:1 (n = 100), 1:1:1 (n = 18), 2:2:1 (n = 1), 3:1:1 (n = 20), 4:1:1 (n = 6), 5:1:1 (n = 63), 5:5:1 (n = 13), 1:1:1:1 (n = 23), 5:1:1:1 (n = 2), 4:1:1:1; (n = 1), 3:1:1:1 (n = 1), 2:1:1:1 (n = 1), 4:3:2:1 (n = 6), 3:2:1:1 (n = 5), 3:3:2:2 (n = 5), and 2:2:1:1 (n = 6). The ratios for purposeful mixtures amplified for 31 cycles included 1:1 (n = 52), 2:1 (n = 43), 3:1 (n = 8), 4:1 (n = 94), 5:1 (n = 2), 1:1:1 (n = 20), 2:2:1 (n = 2), 3:1:1 (n = 21), 4:1:1 (n = 3), 5:1:1 (n = 61), 5:5:1 (n = 17), 1:1:1:1 (n = 14), 2:1:1:1 (n = 2), 3:1:1:1 (n = 3), 4:1:1:1 (n = 3), 5:1:1:1 (n = 3), 4:3:2:1 (n = 6), 3:2:1:1 (n = 6), 3:3:2:2 (n = 7), and 2:2:1:1 (n = 6).

Regarding samples generated from touched items, 53 samples were amplified with HT-DNA amounts and 64 samples with LT-DNA amounts.

### Separation

Amplification products were separated by capillary electrophoresis on an Applied Biosystems 3130*xl* Genetic Analyzer using 0.375 µL per of GeneScan^®^ 500 LIZ^®^ Size Standard (Applied Biosystems) per injection and HIDI formamide (Applied Biosystems). Samples amplified for 31 cycles (LT-DNA) were prepared with 5 µL of PCR product in a total volume of 50 µL, and were injected with 1kV for 22 seconds, 3 kV for 20 seconds or 6kV for 30 seconds as needed (19). Samples amplified using Identifiler^®^ 28 cycles were prepared with 3 µL of PCR product in a total volume of 30 µL and were injected with 1 kV for 22 seconds or 5kV for 20 seconds.

### Data collection

Data were collected with non-variable binning and analyzed using Applied Biosystems GeneScan^®^ and Genotyper^®^ or GeneMapper^®^ software with a 75 relative fluorescence units threshold, a 251 baseline window size, a 10% general filter, which removes peaks that are less than 10% of the highest peak at a locus, and the laboratory’s standard locus-specific stutter filters for Identifiler^®^ 28 cycles and for Identifiler**^®^** 31 cycles. If multiple injections of a given PCR product were generated for a sample, for each locus the injection or amplification that showed the greatest number of labeled peaks that were not off scale or over saturated was used.

### Data analysis

An allele was considered to repeat if it was labeled in 2 out of 2 replicates for HT-DNA samples and in 2 out of 3 replicates for LT-DNA samples. Samples were deemed inconclusive if fewer than 8 labeled repeating peaks over 4 STR loci for HT-DNA samples or 6 STR loci for LT-DNA samples were detected. For all other samples, the DNA profiles of each contributor were compared and the number of missing alleles was noted. For LT-DNA samples, only those loci that showed repeating alleles were used for comparison.

Studies have shown that replication serves to identify allele drop-out and drop-in (24-26). Therefore, the number of repeating alleles at each locus as well as over all loci, and the number of loci with 4 or more, 5 or more, or 6 or more repeating alleles were recorded. Moreover, the number of different alleles at each locus over all replicates, as well as the number of different alleles over all loci and replicates, and the number of loci with 4 or more, 5 or more, or 6 or more different alleles over all replicates were noted. All loci regardless of the presence or absence of repeating alleles were included in the allele counts. Allele counting was done using our own Perl programs (*www.perl.org*).

### Expected number of different alleles

The distribution of the number of different alleles expected from the purposeful mixtures was determined by creating conceptual mixtures using the same donors. Without allowing drop-out or drop-in, the number of different alleles observed in each of the conceptual mixtures was determined. Because some combinations of donors were used for more than one purposeful mixture, there were fewer conceptual mixtures than actual mixtures.

### Mixture ratios

To investigate the effect of varying mixture ratios on the number of different alleles detected, samples were divided into mixtures with similar contributions from all donors and samples with more extreme mixture ratios. For two-person samples, 1:1 and 2:1 mixtures were grouped together (n = 84 LT-DNA; n = 95 HT-DNA) and compared to samples with more extreme mixture ratios (n = 100 LT-DNA; n = 104 HT-DNA). For three- or four-person samples, mixtures with the smallest contributor having at least a 20% contribution were grouped together. For three-person samples, these ratios were 1:1:1, 2:2:1, and 3:1:1 (n = 39 LT-DNA; n = 43 HT-DNA). More extreme ratios included 4:1:1, 5:1:1, and 5:5:1 (n = 82 LT-DNA; n = 81 HT-DNA). For four-person samples, the similar ratios were 1:1:1:1, 3:3:2:2, and 2:1:1:1 (n = 29 LT-DNA; n = 23 HT-DNA), while the more extreme ratios were 3:1:1:1, 4:1:1:1, 5:1:1:1, 4:3:2:1, 3:2:1:1, and 2:2:1:1 (n = 21 LT-DNA; n = 27 HT-DNA).

## Results

### Expected number of different alleles

**Figure 1:**
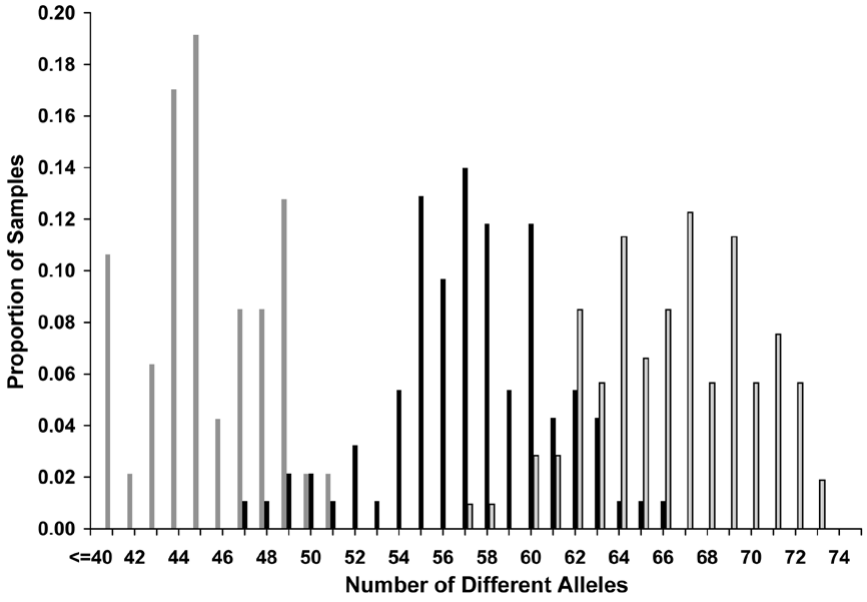
The expected number of different alleles for two-, three-, and four-person mixtures. Assuming no allele dropout, the expected numbers of different alleles for the mixtures tested in this study were enumerated. Since the DNA of the individual donors was combined with various ratios and template amounts, the total number of combinations was 206 where n = 27 for two-person mixtures (gray columns), n = 105 for three-person mixtures (black columns), and n = 43 for four-person (gray columns with black outline) mixtures.

Although there was some overlap between the distribution of the total number of different alleles expected for two-person and three-person mixtures and for three-person and four-person mixtures, there were some distinctions (Figure 1 and Table 1). These results suggest that samples with 49 or fewer alleles are best described as two-person mixtures, with 52 to 59 alleles as three-person mixtures, and with 65 or more alleles as four-person mixtures. However, using this parameter alone, when the number of different alleles falls between 50 and 51 or 60 and 64, one cannot ascertain the number of contributors.

**Table 1:** The range, mean, and standard deviation of the number of different alleles expected in two-, three-, and four-person (p) mixtures

Mixture type	N	Maximum	Minimum	Mean	Standard deviation (SD)	Mean -2 SD	Mean +2 SD
**2p**	57	51	37	45.19	3.19	38.81	51.58
**3p**	105	66	47	57.23	3.68	49.86	64.59
**4p**	109	75	57	66.55	3.75	59.05	74.05

### Observed mixture characteristics

In order to explore additional characteristics of two-, three-, and four-person mixtures, 728 mixtures of varying template amounts, and numbers and ratios of contributors were examined. In this data set, 9 characteristics distinguished three-person from two-person mixtures and 8 characteristics distinguished four-person from three-person mixtures (Table 2). There was one exception as one LT-DNA two-person mixture had 8 loci with 4 or 5 different alleles. The additional alleles could be attributed to stutter as they were in stutter positions of alleles from known contributors.

**Table 2:** Characteristics of three- and four-person high template DNA (HT-DNA) and low template DNA (LT-DNA) mixtures*

>2 Persons	>3 Persons
≥2 loci with ≥5 repeating alleles	≥2 loci with ≥7 repeating alleles
≥2 different loci with ≥5 alleles in one replicate (HT-DNA)	≥3 loci with ≥6 repeating alleles
≥6 (LT-DNA) or 8 (HT-DNA) loci with ≥4 repeating alleles	≥6 loci with ≥5 repeating alleles
1 locus with ≥5 repeating alleles and ≥1 (HT-DNA) or 2 (LT-DNA) other loci with ≥5 different alleles	≥12 (HT-DNA) or 13 (LT-DNA) loci with ≥4 repeating alleles
≥1 locus with 7 different alleles	≥2 loci with ≥7 different alleles
≥2 loci with 6 different alleles	≥3 (HT-DNA) or 5 (LT-DNA) loci with ≥6 different alleles
1 locus with 6 different alleles and ≥3 loci with 5 different alleles (LT-DNA)	≥7 (HT-DNA) or 8 (LT-DNA) loci with ≥5 different alleles
≥4 (HT-DNA) or 5 (LT-DNA) loci with ≥5 different alleles	≥13 loci with ≥4 different alleles
≥8 loci with ≥4 different alleles*	not applicable

*The number of loci with a particular number of repeating and/or different alleles observed in 3 but not 2 person mixtures and in 4 but not 3 person mixtures are listed. Criteria are specific for both HT-DNA and LT-DNA mixtures unless otherwise indicated. Note that one LT-DNA two-person mixture had 8 loci with 4 or 5 different alleles. The additional alleles could be attributed to stutter.

### Observed number of repeating alleles

To determine whether the empirical data supported theoretical predictions for the number of different alleles, the same 728 mixtures were examined. If a low amount of template DNA is amplified, an expected allele consistent with one from a contributor may not be detected, or may drop out. Alternatively, an allele not consistent with the DNA of the contributors may be seen or may drop in. As replication can identify these phenomena, the number of labeled repeating alleles observed was first measured for both HT-DNA and LT-DNA mixtures (data not shown). However, both sample sets displayed significant overlap among the mixture categories such that it was not feasible to delineate any distinct separations.

### Observed number of different alleles

**Figure 2:**
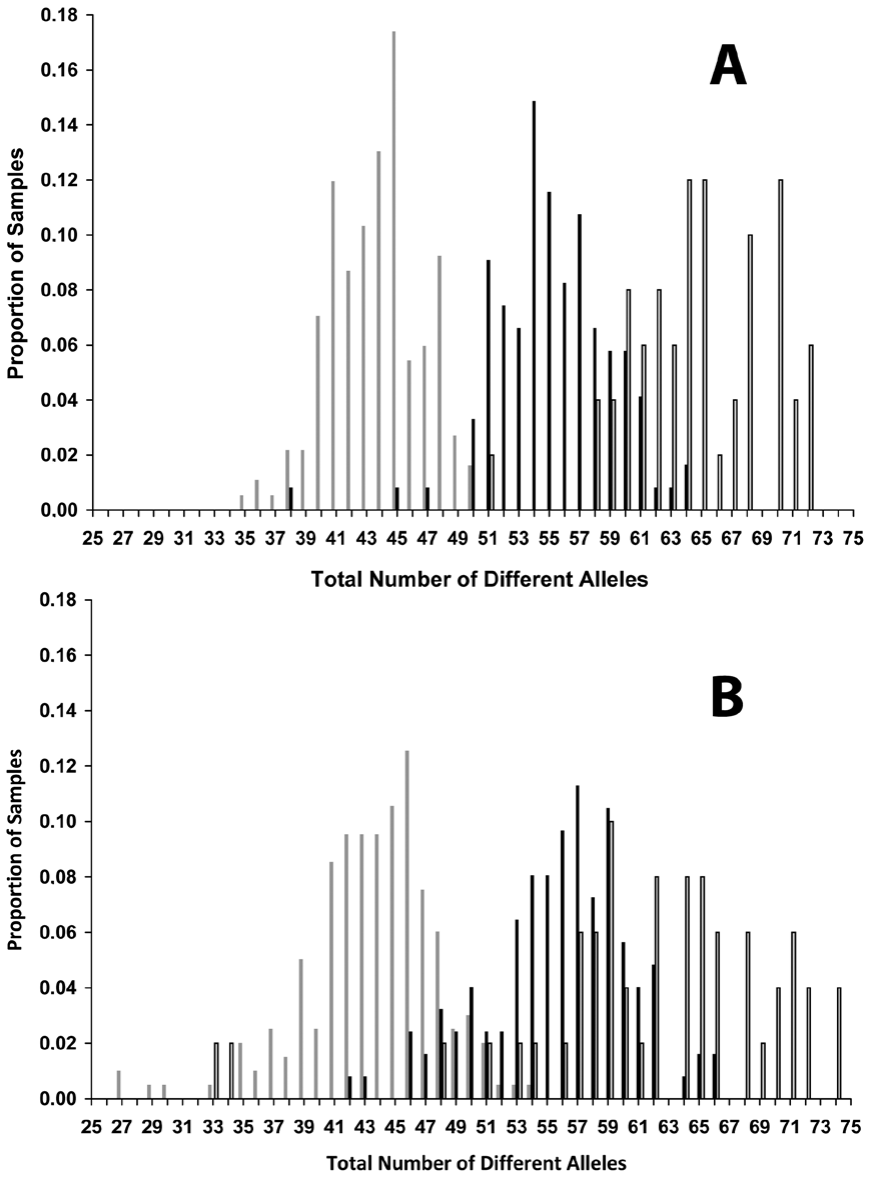
The proportion of samples with different alleles observed over all replicates for purposeful two- (gray columns), three- (solid black) and four- (gray with black outline) person mixtures amplified with high template DNA (HT-DNA) (**A**) and low template DNA (LT-DNA) (**B**) amounts. The total number of different autosomal alleles labeled over 2 HT-DNA or 3 LT-DNA replicates was measured for 728 purposeful mixtures of varying ratios and contributors. For HT-DNA samples, n = 184 for two-person, n = 121 for three-person, and n = 50 for four-person mixtures. For LT-DNA samples, n = 199 for two-person, n = 124 for three-person, and n = 50 for four-person mixtures.

**Figure 3:**
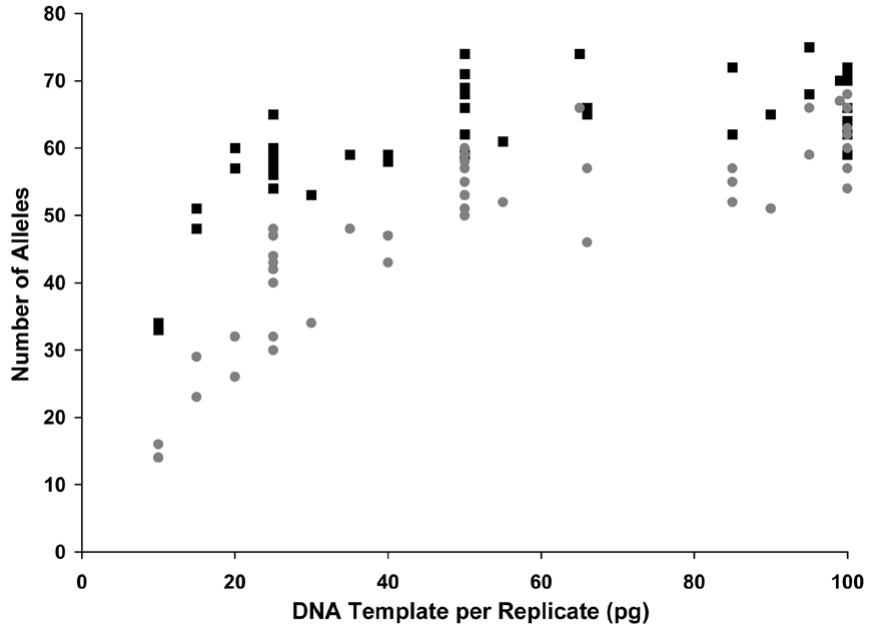
The effect of the amount of template DNA amplified on the number of repeating and different alleles detected. The number of repeating (gray circles) and different (black squares) alleles labeled for each of 50 four-person low template DNA (LT-DNA) purposeful mixtures tested are shown with the corresponding amount of DNA amplified in each of 3 replicates. These values vary dramatically depending upon the template amount for samples with less than 50 pg.

The number of labeled different alleles was examined for HT-DNA (Figure 2A) and LT-DNA (Figure 2B) mixtures. These data provided more divisions between samples sets than the number of repeating alleles. The number of different alleles seen for four-person mixtures increased with the amount of template DNA amplified for samples with up to 50 pg of template DNA (Figure 3). Although less extreme, this was also the case for two- and three-person mixtures (data not shown). Therefore, each data set was subdivided into two groupings representing the upper and lower quantitative ranges of HT-DNA and LT-DNA mixtures. Specifically for HT-DNA samples, these ranges were 250 pg and above and 150 pg to 250 pg, and for LT-DNA samples the ranges were 50 pg and above and 10 pg to 50 pg.

Results are enumerated for HT-DNA (Table 3) and LT-DNA (Table 4) mixtures. In order to establish recommended thresholds to differentiate two-, three-, and four-person mixtures, the observed as well as the expected results were both evaluated (Table 5) in two manners. First, to estimate cut off values for two-person mixtures for example, the mean plus 2 standard deviations for two-person mixtures and the mean minus 2 standard deviations for three-person mixtures were used. A second set of thresholds was made using the maximum and the minimum values. Lastly, recommended thresholds were established considering the means, the extreme values, characteristics of the outliers, as well as theoretical expectations.

**Table 3:** The range, mean, and standard deviation of the number of different alleles observed in two-, three-, and four-person (p) mixtures amplified with high template DNA (HT-DNA) amounts

Type and template amount	N	Maximum	Minimum	Mean	Standard deviation (SD)	Mean -2 SD	Mean +2 SD
**2p less than 250 pg**	75	50	35	43.53	3.21	37.11	49.95
**3p less than 250 pg**	56	64	50	55.00	2.97	48.06	60.94
**4p less than 250 pg**	14	70	58	64.00	4.10	55.80	72.20
**2p 250 pg and above**	109	50	37	43.87	2.86	38.15	49.59
**3p 250 pg and above**	65	64	38	55.40	4.40	46.60	64.20
**4p 250 pg and above**	36	72	51	65.03	4.66	55.71	74.35
**2p all HT-DNA**	184	50	35	43.73	3.01	37.71	49.75
**3p all HT-DNA**	121	64	38	55.21	3.80	47.61	62.81
**4p all HT-DNA**	50	72	51	64.74	4.45	55.84	73.64

**Table 4:** The range, mean, and standard deviation of the number of different alleles observed in two-, three-, and four-person (p) mixtures amplified with low template DNA (LT-DNA) amounts*

Type and template amount	N	Maximum	Minimum	Mean	Standard deviation (SD)	Mean -2 SD	Mean +2 SD
**2p less than 50 pg**	90	51	27	42.66	4.55	33.56	51.76
**3p less than 50 pg**	41	66	42	53.00	5.39	42.23	63.77
**4p less than 50 pg**	14	65	53	58.43	3.39	51.65	65.21
**2p 50 pg and above**	99	54	27	44.77	3.68	37.41	52.13
**3p 50 pg and above**	83	66	47	57.08	3.62	49.84	64.32
**4p 50 pg and above**	30	75	59	66.80	4.53	57.74	75.86
**2p all LT-DNA**	189	54	27	43.76	4.24	35.28	52.24
**3p all LT-DNA**	124	66	42	55.73	4.68	46.37	65.09
**4p all LT-DNA**	44	75	53	64.14	5.73	52.68	75.60

*Values for 10 pg, 15 pg, and 20 pg mixtures were not included as there were no purposeful three-person mixtures measured with those template amounts**.**

**Table 5:** Observed, expected, and the recommended ranges of the number of different alleles in two-, three-, and four-person (p) high template DNA (HT-DNA) and low template DNA (LT-DNA) mixtures

Groupings	2p	2p-3p	3p	3p-4p	4p
**Recommended LT-DNA<50 pg**	≤46	47-51	52-56	57-66	≥67
**Recommended LT-DNA≥50 pg**	≤46	47-54	55-56	57-66	≥67
**Recommended HT-DNA**	≤46	47-51	52-56	57-66	≥67
Expected mean ±2 SD*	≤49	50-51	52-59	60-64	≥65
**Expected max and min**	≤46	47-51	52-56	57-66	≥67
**<50 pg mean ±2 SD**	≤42	43-51	none	52-64	≥65
**<50 pg max and min**	≤41	42-51	52	53-66	≥67
**50-100 pg mean ±2 SD**	≤49	50-52	53-57	58-64	≥65
**50-100 pg max and min**	≤46	47-54	55-58	59-66	≥67
**<250 pg mean ±2 SD**	≤48	49	50-55	56-60	≥61
**<250 pg max and min**	≤49	50	51-57	58-64	≥65
**≥250 pg mean ±2 SD**	≤46	47-49	50-55	56-64	≥65
**≥250 pg max and min**	≤37	38-50	none	51-64	≥65

*SD – standard deviation.

### HT-DNA

The maximum, minimum, and the average plus or minus 2 standard deviations of the number of different alleles observed in all HT-DNA samples can help distinguish among two-, three-, and four-person samples (Table 3). Two 5:1:1 260 pg three-person samples contained 45 or 38 different alleles, values below the average minus 2 standard deviations. Neither sample met the criteria for three-person mixtures regarding the number of loci with 4 or more repeating or different alleles. Moreover, for both of these samples, 3 or more alleles belonging to 1 or more of the sample donors were not labeled. Due to allelic drop-out these samples may be better described as two-person rather than as three-person mixtures.

Although in this data set no more than 50 different alleles were labeled for any of the two-person mixtures tested, theoretically it was possible to generate 51 alleles from one combination of 2 of the donors. Using all data sources, the presence of 52 or more different alleles in HT-DNA mixtures indicated that the sample consisted of more than 2 contributors. Forty-six or fewer different alleles suggested that the sample could best be described as originating from 2 individuals. These groupings signify that samples containing 47, 48, 49, 50, or 51 alleles should be examined for the additional criteria in Table 2.

For HT-DNA three-person mixtures, at most 64 alleles were seen, although 1 combination of 3 donors consisted of 66 different alleles. Therefore, 67 or more different alleles signified a four-person HT-DNA mixture. Using the cutoffs from Table 5 and the guidelines presented in Table 2, 116 of the 121 HT-DNA three-person mixtures met said criteria, giving an overall success rate of 96%. Forty of the 121 HT-DNA three-person mixtures had at least 1 true contributor for which 3 or more alleles were missing. If only the remaining 81 samples are considered to be true three-person mixtures, the success rate increases to 97.5%, as only 2 of these samples were deemed to be two-person mixtures by the cutoffs and the Table 2 guidelines.

Only 1 HT-DNA purposeful four-person mixed sample showed 51 different alleles. Two of the 4 donors to this 400 pg 2:2:1:1 experienced significant drop-out, with 7 and 11 missing alleles. Thus, this sample may be better described as a three-person than a four-person mixture. Although in this study all other four-person mixtures amplified had 58 or more different alleles, 1 combination of 4 donors was composed of 57 different alleles. Therefore, the presence of 52-56 different alleles suggested a three-person mixture while 57-66 different alleles warranted additional scrutiny.

Following these guidelines, 7 or 14% of the 50 HT-DNA four-person mixtures assessed better resembled three-person mixtures than four-person mixtures. Each of these 7 samples had 61 or fewer different alleles and would fall within the zone encompassing both three-and four-person mixtures. Furthermore, these samples did not demonstrate the characteristics listed in Table 2 for four-person mixtures. If samples with at least 1 contributor missing 3 or more alleles are not included in these counts, as these samples may be better described as three-person than four-person mixtures, 6 out of 44 samples (again, 14%) would be estimated to have 3, not 4, contributors.

### LT-DNA

Considering the estimated ranges, as well as the maximum and the minimum numbers of different alleles observed, LT-DNA samples had less distinct divisions, likely due to allelic drop-out. For example, the lowest expected number of different alleles from the combinations of 3 donors tested was 47 although some three-person samples were observed to have fewer than 47 different alleles (Table 4). Nevertheless, samples were for the most part accurately triaged based on guidelines for the number of loci with 4 or more repeating and/or different alleles.

In fact, the 25pg 5:1:1 mixture, which had the minimum number of alleles, 42, did not meet the Table 2 criteria for a three-person mixture as it did not have any loci with 5 or more repeating alleles and only had 1 locus with 4 different alleles and 1 locus with 5 different alleles. Since 2 of the 3 contributors were missing at least 3 alleles each, this mixture may be better described as two-person than three-person. Similarly, 4 other purposeful 25 pg 5:1:1 mixtures that had either 43 or 46 different alleles did not meet the qualitative criteria for three-person mixtures, and at least 1 donor for each was missing 3 or more alleles.

On the other hand, another 25 pg 5:1:1 mixture had 48 different alleles. Unlike the previous samples, this mixture had 7 loci with 4 different alleles and 1 locus with 5 different alleles, both of which are qualities of three-person mixtures. As none of the contributors were missing more than 2 alleles, this mixture is probably best described as three-person in spite of the low total allele count. Accounting for all data, samples with 52 or more alleles could have more than 2 contributors, whereas samples containing 47-51 different alleles should be further evaluated.

Regarding LT-DNA two-person mixtures with 50 pg or more, samples with 55 or more alleles contained more than 2 contributors and values from 47-54 should be assessed for other characteristics. Using the cutoffs in Table 5 and the guidelines in Table 2, 104 of the 124 LT-DNA three-person mixtures qualified as such, giving an overall success rate of 84% for these samples. As with the HT-DNA samples, about 1/3 had 1 or more contributors for which at least 3 alleles were missing. If only the 80 LT-DNA three-person samples with no contributors missing more than 2 alleles are considered to be true three-person mixtures, the success rate climbs to 95%, as only 4 of these samples resembled two-person mixtures by the cutoffs and the Table 2 guidelines.

To distinguish between three-person and four-person mixtures, the upper end of the distribution of the number of different alleles seen in the three-person samples and the lower end of the distribution of the number of different alleles seen in the four-person mixtures can be used. Sixty-seven or more different alleles signified a four-person LT-DNA mixture. For samples with less than 50 pg, all values from 52-56, and for samples with 50 pg or more, values of 55 or 56, suggested 3 contributors. Samples with 57-66 different alleles should also be evaluated with the Table 2 guidelines.

Overall, 25 of the 50 LT-DNA four-person samples looked like four-person samples by the total number of different alleles labeled and/or by the patterns listed in Table 2. However, the vast majority of the samples that did not meet the four-person criteria contained less than 50 pg of template DNA. Mixtures with template amounts of 10 pg and 15 pg made with DNA from 4 contributors contained 33-51 different alleles suggesting that there was so much drop-out that they were actually composed of fewer than 4 persons. Accordingly, the mixtures did not meet the qualitative criteria for four-person mixtures, and some of the contributors were missing more than 2 alleles each, providing support that the guidelines enumerated reflect the number of fully-represented contributors to mixtures. It should be noted that these samples were not included in Tables 4 and 5 as template amounts less than 25 pg were not amplified for three-person mixtures.

Sixteen four-person samples were amplified with 20 to 40 pg of template DNA. None of these samples showed more than 66 different alleles and only 2 would be called four-person mixtures by the criteria in Table 2. At least one contributor to 15 of the 16 samples was missing more than 2 alleles and, thus, these samples may be better described as three-person samples than as four-person samples.

Four-person mixtures with at least 50 pg of template DNA presented fewer challenges. None of the 4 contributors to 24 of the 30 four-person mixtures with 50 pg or more of template DNA were missing more than 2 alleles. Of these 24 samples, 18 (75%) met the four-person mixture criteria of 67 or more different alleles and/or the characteristics shown in Table 2. If all samples with at least 50 pg of DNA were considered, regardless of whether or not any contributors’ alleles were missing, 23 out of 30 (77%) met the four-person mixture criteria. Thus, the success rate for four-person LT-DNA samples with at least 50 pg of template DNA was 75%-77%.

### Mixture ratios

The purposeful mixtures in this study included mixture ratios of varying extremes; therefore, samples showed different degrees of allelic drop-out, as measured by the number of different alleles detected compared to the number of different alleles expected. In order to determine whether varying mixture ratios were masking any important trends, samples were divided into two categories: those with similar contributions from all donors and those with more extreme mixture ratios.

**Figure 4:**
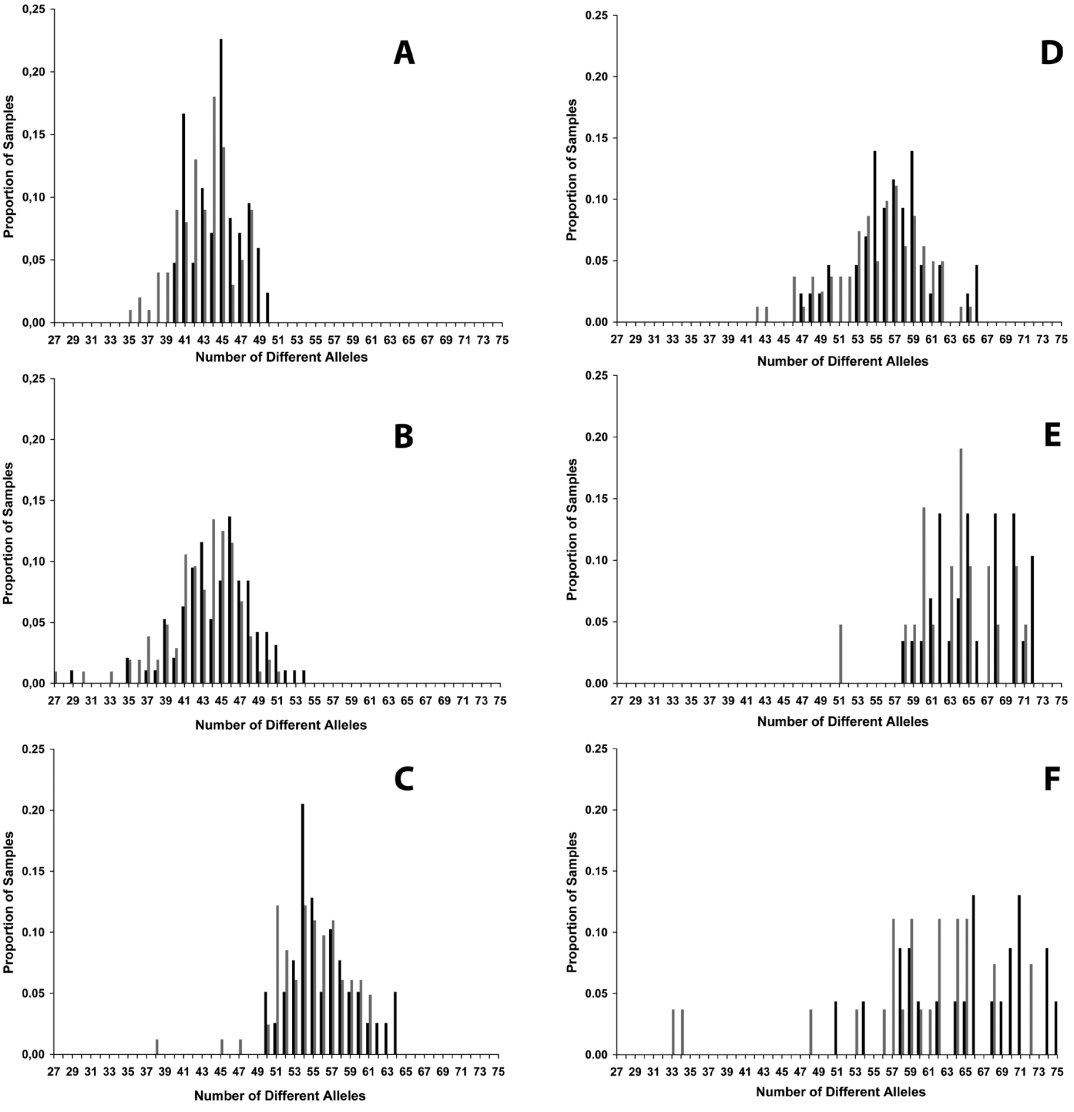
The distribution of the number of different alleles is comparable for mixtures with similar contributions from all donors and for more extreme mixture ratios. Figures (**A**) and (**B**) show the number of different alleles observed in two-person samples with 1:1 or 2:1 mixture ratios (black columns) and more extreme ratios (gray columns) in high template DNA (HT-DNA) and low template DNA (LT-DNA) samples, respectively. Figures (**C**) and (**D**) show the number of different alleles observed in three-person samples with 1:1:1, 2:2:1, and 3:1:1 mixture ratios (black columns) and more extreme ratios (gray columns) in HT-DNA and LT-DNA samples, respectively. Figures (**E**) and (**F**) show the number of different alleles observed in four-person samples with 1:1:1:1, 3:3:2:2, or 2:1:1:1 mixture ratios (black columns) and more extreme ratios (gray columns) in HT-DNA and LT-DNA samples, respectively. The number of samples in each category is as follows: (**A**) HT-DNA two-person mixtures with similar ratios: n = 84, with extreme ratios: n = 100; (**B**) LT-DNA two-person mixtures with similar ratios: n = 95, with extreme ratios: n = 104; (**C**) HT-DNA three-person mixtures with similar ratios: n = 39, with extreme ratios: n = 82; (**D**) LT-DNA three-person mixtures with similar ratios: n = 43, with extreme ratios: n = 81; (**E**) HT-DNA four-person mixtures with similar ratios: n = 29, with extreme ratios: n = 21; (**F**) LT-DNA four-person mixtures with similar ratios: n = 23, with extreme ratios: n = 27.

The distribution of number of different alleles observed in LT-DNA and HT-DNA two-, three-, and four-person mixtures was not substantially different for similar ratios and for more extreme ratios (Figures 4A through 4F). The very low observations in Figure 4F, the LT-DNA four-person samples, occurred in the 10 pg and 15 pg samples, which are likely to experience extreme drop-out regardless of mixture ratio.

### Testing with touched items

**Figure 5:**
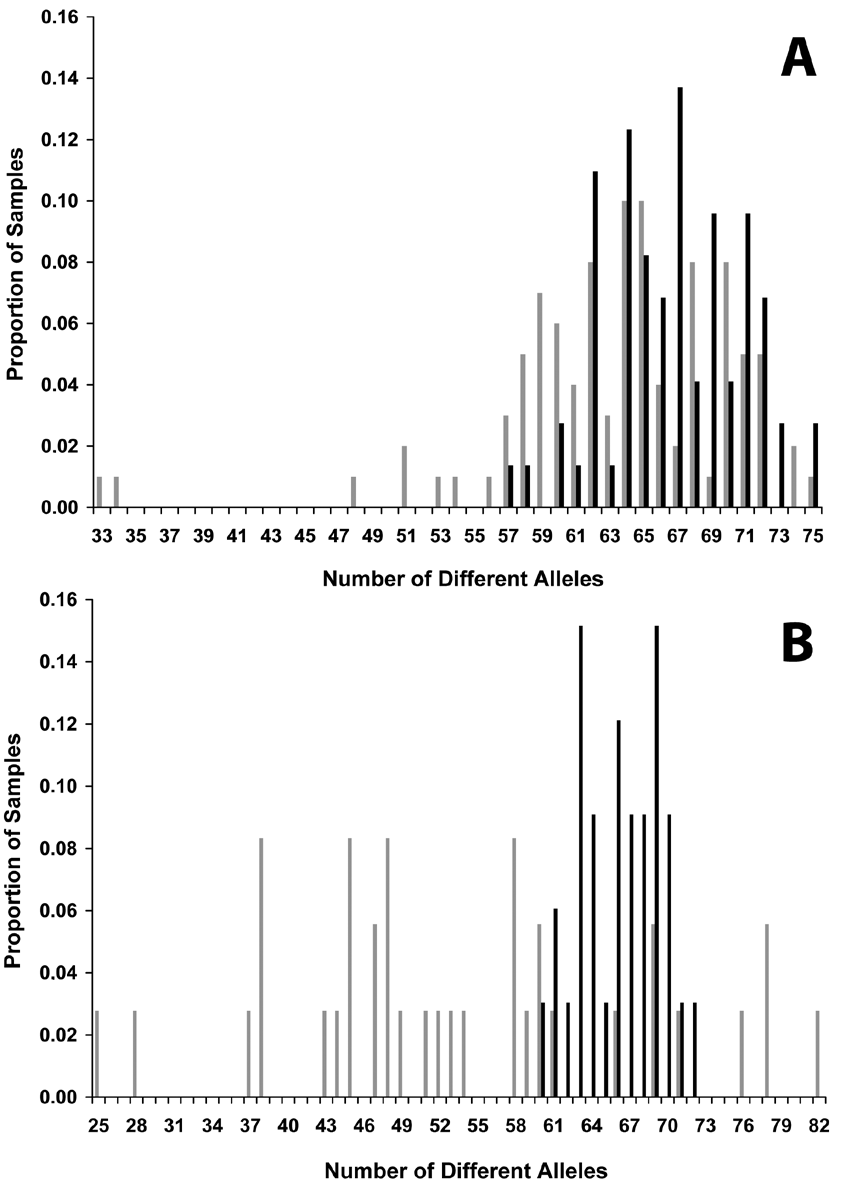
The distribution of the number of different alleles is more variable for touched items than for purposeful mixtures. Figure (**A**) shows the number of different alleles observed (gray columns) and expected (black columns) in 100 four-person mixtures. Figure (**B**) illustrates the number of different alleles observed (gray columns) and expected (black columns) in 36 samples generated from items handled consecutively by 4 individuals.

Samples generated from items handled by 2, 3, or 4 contributors were evaluated with the derived guidelines. These non-purposeful mixtures included another variable, the shedder status of the individuals who touched the items. Therefore, the distribution of the observed number of different alleles in four-person purposeful mixtures more closely approximated the expected distribution for four-person mixtures (Figure 5A) than for items touched by 4 individuals (Figure 5B). This trend was evident with two- and three-person mixtures as well (data not shown). Due to variability in shedding as well as in DNA recovery, the precise composition of the DNA extract and the number of contributors originally amplified are unknown. For example, it is very likely that for some items touched by 4 persons, DNA from only 2 contributors was amplified; therefore, precise accuracy rates for estimating the number of contributors cannot be calculated.

## Discussion

The observed results did not precisely reflect the expected number of different alleles without drop-out or drop-in. Due to the nature of a sample, degradation, the mixture ratio or the number of contributors to a mixture, allele(s) may not be amplified or may drop out, reducing the total number of different alleles observed. Alternatively, an allele that is not consistent with those of the known contributors may “drop in.” This would increase the number of different alleles seen (24-26).

All loci were evaluated in this study, but the total number of alleles observed or the power of discrimination at any particular locus was not considered. However, it was noted that 5 alleles were labeled in three-person mixtures more frequently at D2S1338, D19S433, D18S51, FGA, and vWA than at the other 10 loci (data not shown). Three of these loci also had a high propensity for allelic dropout (19). Therefore based on these results, the use of individual loci to distinguish the number of contributors was not helpful.

Overall, the samples with numbers of different alleles within the two-, three-, or four-person mixture zones resembled two-, three-, or four- person mixtures by the Table 2 criteria. There were a few samples that were created with DNA from 3 donors, for example, that had numbers of different alleles in the two-person range. These samples did not meet the criteria for three-person mixtures presented in Table 2, suggesting that the estimates of the number of contributors by the criteria and by the total allele count are consistent.

Extensive allele sharing could also cause a three-person mixture to appear as a two-person mixture (10,11). For example, a 65 pg 5:1:1 mixed sample with 47 different alleles was within the “gray zone” in which the two-person and three-person samples overlap. This sample did not meet the criteria for a three-person mixture as only 1 locus had 5 repeating alleles and 5 loci had 4 different alleles. All 3 donors whose DNA were used to create this sample could be positively associated to the mixture in that none of the donors had more than 2 missing alleles. However, although these samples were collected randomly, based on kinship analysis using the CODIS 5.7.4 Popstats program, it is possible that the major contributor and one other contributor could be brothers.

There was one instance wherein a two-person mixture appeared to be a three-person mixture. This 50 pg mixture had 54 different alleles, a number well beyond what is expected for a two-person mixture. Further investigation of this sample revealed that it contained 4 loci with 5 different alleles and 4 loci with 4 different alleles; thus, this sample met the Table 2 criteria for a three-person mixture. However, the donors to this mixture had only 49 different alleles between them and the extra alleles were always in the stutter position and did not repeat. Thus, factors such as the contribution of stutter should also be considered when estimating the number of contributors to a sample. Interestingly, this sample is the only two-person mixture for which at least 8 loci had at least 4 different alleles, a guideline which was a critical parameter to capture true three-person mixtures with in-between numbers of different alleles.

Using the total number of different alleles and the characteristics listed in Table 2, 86% of all HT-DNA and 75%-77% of 50-100 pg LT-DNA four-person purposeful mixtures resembled four-person mixtures. This was not the case for almost all of the samples with less than 50 pg of template DNA. When the profiles of the true contributors were examined, the very low template samples showed extreme drop-out and could better be described as three-person or even two-person mixtures.

In summary, including only samples for which none of the true contributors were missing more than 2 alleles, 99%, 96%, and 82% of two-, three-, and four- person purposeful mixtures, respectively, met the appropriate criteria for the number of contributors. Samples that did not, exhibited extreme phenomena, such as excessive allele sharing or stutter. The success rates stated here are data driven estimates and may not be representative of all forensic samples. Although a variety of template amounts and mixture ratios were included in this sample set, forensic samples may exhibit qualities not observed in these mixtures.

Mixtures generated from items handled by two-, three-, or four-persons more closely mimicked evidentiary samples. Results mirrored the purposeful mixtures except that more alleles that were consistent with the individuals who touched the items were not amplified and more alleles that were foreign to these individuals were detected. The absence of alleles could be attributed to the fact that individuals shed to varying degrees and thus if a strong shedder is the last person who handled an item, DNA from the first person may not be recovered (27). Alleles foreign to the donors could be from DNA that was already on the items as some of the items used in the study were not cleaned prior to handling to mimic real situations. Consequently, touched items displayed a wider range of the number of different alleles than purposeful mixtures indicating that there was more allele drop-out and drop-in.

Thus, it is more challenging to accurately estimate the number of contributors to samples from touched items than to samples from body fluids. Nevertheless, general ranges for the number of different alleles are still indicative of the number of contributors. When these values are within the intermediate ranges for categories, empirically defined characteristics have proven useful. In a few instances, extreme allele sharing, the allele sharing inherent to four-person mixtures, or the lack of sharing coupled with increased stutter could mask the true number of contributors.

A probabilistic model involving allele frequencies and the probability of drop-out and drop-in could address these issues (28-30). For example, the probability that a three-person mixture would show 42 alleles and not display any of the characteristics shown in Table 2 could be assessed. However, a probabilistic approach will also have limitations as classifications will depend upon estimated drop-out and drop-in parameters. Although a mixture may be composed of 3 contributors, only 2.5 contributors may be apparent, suggesting that the true number of contributors is a continuum. Depending upon the values of the drop-out and drop-in parameters, such a sample may be classified as a three-person mixture with drop-out or a two-person mixture with drop-in. Although all classifications in general are always estimates, the empirically based guidelines described herein provide a useful tool to approximate the number of contributors to mixtures in forensic casework.
